# Lung cancer after kidney transplantation: a 50-year experience at a single institution

**DOI:** 10.1007/s00595-024-02819-9

**Published:** 2024-03-28

**Authors:** Hiroki Watanabe, Yuka Kadomatsu, Shuhei Hakiri, Hiromu Yoshioka, Takahisa Hiramitsu, Kenta Futamura, Manabu Okada, Norihiko Goto, Shunji Narumi, Yoshihiko Watarai, Toyofumi Fengshi Chen-Yoshikawa

**Affiliations:** 1 Department of Thoracic Surgery, Nagoya University Graduate School of Medicine, 65 Tsurumai-Cho, Showa-Ku, Nagoya, 466-8550 Japan; 2 Department of Thoracic Surgery, Japanese Red Cross Aichi Medical Center Nagoya Daini Hospital, 2-9 Myoken-Cho, Syowa-Ku, Nagoya, 466-8650 Japan; 3 Department of Transplant and Endocrine Surgery, Japanese Red Cross Aichi Medical Center Nagoya Daini Hospital, Nagoya, Japan

**Keywords:** Lung cancer, Kidney transplantation, Thoracic surgery, Thoracic malignancies

## Abstract

**Purpose:**

To investigate the clinical characteristics of lung cancer that develops after kidney transplantation.

**Methods:**

The clinical data of patients with lung cancer diagnosed after kidney transplantation were collected retrospectively. The medical records were extracted from our database. All patients underwent routine chest examination after kidney transplantation.

**Results:**

In total, 17 lung tumors were detected in 15 (0.6%) of 2593 patients who underwent kidney transplantation at our institution. Eleven lung tumors were completely resected from a collective 10 patients (surgical group). The remaining five patients did not receive surgical treatment (nonsurgical group). The surgical group underwent wedge resection (*n* = 5), segmentectomy (*n* = 1), lobectomy (*n* = 3), and bilobectomy (*n* = 1). The pathological stages were 0 (*n* = 1), IA1 (*n* = 2), IA2 (*n* = 4), IA3 (*n* = 2), and IB (*n* = 1). The surgical group had a significantly better prognosis than the nonsurgical group. There were no perioperative complications related to kidney transplantation in either group.

**Conclusions:**

Routine chest examination would be useful for the early diagnosis and treatment of lung cancer after kidney transplantation. Moreover, surgical resection for early-stage lung cancer was associated with a better prognosis for kidney transplantation patients.

## Introduction

Kidney transplantation for end-stage kidney disease is superior to dialysis in terms of quality and quantity of life and cost. Immunosuppressive agents are used to prevent graft failure after kidney transplantation; however, immunosuppression is associated with the development of malignant tumors [1]. Moreover, data on the development of lung cancer after kidney transplantation in patients who are immunosuppressed are limited. Conversely, with technological developments in radiological and surgical fields, less-invasive surgical interventions for small lung tumor are being performed worldwide [2, 3]. This retrospective study evaluates the development of lung cancer in patients who underwent kidney transplantation at a single institution and assesses their outcomes with the aim of improving long-term prognosis after kidney transplantation.

## Methods

### Study design and patients

We reviewed the clinical data of patients who underwent thoracic surgery after kidney transplantation at the Japanese Red Cross Aichi Medical Center Nagoya Daini Hospital between June 1972 and December 2021. Prior to thoracic surgery, all patients had received a kidney transplant at the same institution and had been advised to quit smoking before the transplantation. All patients underwent thoracic surgery at the same institution where they were diagnosed with lung cancer. The following data were collected: demographic variables, time from kidney transplantation until lung cancer was detected, type of transplanted kidney, smoking habit, diagnosis of lung cancer, types of immunosuppressive treatment, kidney function, histology of lung cancer, clinical and pathologic stage of the disease, types of treatment, complications, survival time, and cause of death. Patients with insufficient or missing clinical information were excluded from this analysis.

### Post-kidney transplantation follow-up

All patients who underwent kidney transplantation at the Japanese Red Cross Aichi Medical Center Nagoya Daini Hospital or Masuko Memorial Hospital were followed up. They visited the hospital every 2 weeks for the initial 3 months after kidney transplantation, then every 4 or 5 weeks for the subsequent 9 months, and every 6–9 weeks after 1 year. Immunosuppressive agents were administered from 3 months after kidney transplantation, according to one of four different protocols during the maintenance phase (Table 1) [4–6]. If a malignancy developed, treatment with mycophenolate mofetil (MMF) was discontinued, and everolimus was started for patients who received MMF after kidney transplantation. If everolimus was administered after kidney transplantation, treatment was continued after the malignancy was diagnosed. Chest radiography was performed every 6 months and chest computed tomography (CT) scans were performed every 2 years to screen for thoracic malignancies.

**Table 1:** Protocol on the use of immunosuppressive agents (post-kidney transplant maintenance phase)

FK506 + MMF + PSL
CsA + MMF + PSL
CsA + EVR + PSL
FK506 + EVR + PSL

Abbreviations: *FK506* tacrolimus, *MMF* mycophenolate mofetil, *PSL* prednisolone, *CyA* cyclosporine, *EVR*, everolimus

### Staging of lung cancer

Staging of lung cancer was based on the eighth edition of the TNM classification of lung cancer, as proposed by the Union for International Cancer Control and the International Association for the Study of Lung Cancer in 2017.

### Statistical analyses

Descriptive statistics of continuous variables are expressed as the median and categorical variables as the number and percentage. The probability of survival was assessed using the Kaplan–Meier method. Between-groups comparisons were performed using the Mann–Whitney U test for continuous and ordinal variables. *P*-values of ≤ 0.05 were considered significant. All statistical analyses were performed using EZR.R software, version 2.8-0 (Saitama Medical Center, Jichi Medical University, Saitama, Japan).

## Results

Among 2593 patients who underwent kidney transplantation, 2246 received living donor kidney transplants, 315 received cadaveric kidney transplants, 27 underwent simultaneous pancreas–kidney transplantation, and 5 underwent pancreatic transplantation after living donor kidney transplantation. Table 2 summarizes the clinical characteristics of the patients with lung cancer. Lung cancer developed after kidney transplantation in 15 (0.6%) patients (10 men and 5 women, with a median age of 64.0 and an interquartile range of 58.9–74.9 years, at the time of lung cancer diagnosis). Lung cancer was detected on routine chest examination after kidney transplantation in eight patients, by routine chest radiography in two, and by routine chest CT in six. Imaging tests were performed to scrutinize symptoms and other diseases in three patients and lung cancer was discovered incidentally. In one patient, a pulmonary nodule was detected during systemic examination prior to kidney transplantation, and transbronchial lung biopsy was performed. This lung nodule was initially diagnosed as an inflammatory nodule, but after kidney transplantation, the nodule grew, and biopsy was performed again, resulting in a diagnosis of lung cancer. The doses of immunosuppressive agents were reduced after the detection of lung cancer in some patients. In one patient, the dose of MMF was tapered from the time that lung cancer was suspected clinically. It was then discontinued and everolimus was started, but was discontinued because of drug-induced interstitial lung disease, thought to be caused by everolimus. The MMF dose in another patient was reduced by half after lung cancer was diagnosed. He was never treated with everolimus and later died of lung cancer. Patients with up to stage IIIA lung cancer usually underwent surgery. Two patients opted for radiation therapy because of their comorbidities. During the study period, 11 lung tumors in 10 patients were completely resected.

**Table 2:** Characteristics of the patients with lung cancer after kidney transplantation

No.	Age/sex	Duration from KT to lung cancer development (years)	LD/CD	Smoking history	Mode of diagnosis of lung cancer (imaging test)	Clinical TNM (stage)	Histology	Treatment	Pathological TNM (stage)	Survival (months) and cause of death
1	53/F	34.1	LD	–	Routine (CT)	T1miN0M0 (IA1)	Ad	Surgery (wedge resection)​	T1aN0M0 (IA1)	31: alive
2​	63/F​	26.5​	CD​	–	Routine (CT)	T1miN0M0 ​(IA1)	Ad​	Surgery(wedge resection)​	T1miN0M0 ​(IA1)	101: alive​
3​	64/F​	27.1​	CD​	–	Routine (CT)	T1miN0M0 ​(IA1)	Ad​	Surgery (wedge resection)​	TisN0M0​ (0)	36: alive​
4​	61/M​	17.4​	CD​	+	Symptom​s (CT)	T3N0M0 (IIB)	Ad​	Radiation​	–	3: radiation pneumonitis
5​	59/M​	13.7​	CD​	+	Incidental detection (CT)​	T1aN0M0​ (IA1)	Unknown​	Radiation​	–	84: myocardial infarction
6​	64/M​	10.0​	LD​	+	Routine (Xr)	T1bN0M0 (IA2​)	Sq	Surgery (lobectomy)​	T1bN0M0 (IA2)	7: progressive cancer
7​	56/M​	3.3​	LD​	+	Incidental detection​ (Xr)	T2aN3M0 (IIIB​)	Small	Radiation​	–	3: progressive cancer
8​	58/M​	7.9​	LD​	+	Routine (CT)	T1bN0M0 (IA2)​	Ad​	Surgery (wedge resection)​	T1bN0M0 (IA2)	41: alive​
9​	48/F​	3.5​	LD​	+	Routine (CT)​	T1cN0M0 (IA3​)	Sq	Surgery (lobectomy)​	T1cN0M0 (IA3​)	79: alive​
10​	75/M​	4.0​	LD​	+	Routine (Xr)	T2bN1M0 (IIB)	Sq​	Surgery (bilobectomy)​	T1cN0M0 (IA3​)	51: gastrointestinal tract perforation
11​	79/M​	5.7​	LD​	–	Symptoms (CT)​	TXNXM1a (IVA​)	Ad​	BSC​	–	Progressive cancer^a^
12​	73/F​	4.5​	LD​	–	Incidental detection​ (CT)	T1bN0M0 (IA2)​	Ad​	Surgery (segmentectomy)​	T1bN0M0 (IA2)	30: alive​
13	76/M	1.2	LD	+	Systemic scrutiny prior to KT (CT)	T1cN2M0 (IIIA)	Sq	Surgery (lobectomy)	T2aN0M0 (IB)	66: alive
14	81/M	2.7	LD	+	Routine (CT)	T1bN0M0 (IA2)	Sq	Surgery (wedge resection)	T1bN0M0 (IA2)	14: second cancer progression
15	69/M	0.7	LD	+	Symptoms (Xr)	T2aN3M1c (IVB)	Small	Chemotherapy	–	12: progressive cancer

Abbreviations: *KT* kidney transplantation, *LD* living donor, *CD* cadaveric donor, *Xr* X-ray, *Ad* adenocarcinoma, *Sq* squamous cell carcinoma, *small* small cell carcinoma, *BSC* best supportive care

^a^The survival period was not described because of BSC

The surgical approaches included eight endoscopic surgeries and two open thoracotomies. Wedge resection (*n* = 5), segmentectomy (*n* = 1), lobectomy (*n* = 3), and bilobectomy (*n* = 1) were performed. Bilobectomy involved the right middle and lower lobes. Lobectomy involved the right upper, middle, and left lower lobes. Systemic mediastinal lymph node dissection was performed with bilobectomy, lobectomy, and segmentectomy. Patients who underwent lobectomy and bilobectomy, except for one who underwent middle lobectomy, had pericardial fat pad reinforcement at the bronchial stump. In patients with lung cancer who underwent wedge resection or segmentectomy, ^18^F-fluorodeoxyglucose uptake in the hilar or mediastinal lymph nodes was not observed on preoperative positron emission tomography. Concerning the clinical stage, none of the patients had metastasis in the hilar or mediastinal lymph nodes. Peripheral tumors were resected via wedge resection and central tumors were resected via segmentectomy. Segmentectomy involved the left S4–S5. The pathologic stages were 0 (*n* = 1), IA1 (*n* = 2), IA2 (*n* = 4), IA3 (*n* = 2), and IB (*n* = 1).

Table 3 shows the perioperative characteristics of the participants. One patient suffered postoperative bleeding, requiring emergency re-opening of the chest. There were no complications related to the immunosuppressive agents. The median postoperative serum creatinine (Cr) level and estimated glomerular filtration rate (eGFR) after surgery did not differ significantly from those before surgery (Table 4). Postoperatively, even in the immediate perioperative period, none of the patients were reintroduced to dialysis. There were no hospital deaths among patients who underwent surgical resection.

**Table 3:** Perioperative characteristics of the participants

Preoperative serum Cr level (mg/dL)	1.23	(1.17–1.80)
Preoperative eGFR (mL/min/1.73 m^2^)	39.0	(29.7–48.4)
Operative duration (min)	126	(54.6–203.5)
Volume of blood loss (mL)	4	(0–16.3)
Complications, *n*	1	
Postoperative bleeding	1	
Postoperative serum Cr level (mg/dL)	1.18	(1.09–1.47)
Postoperative eGFR (mL/min/1.73 m^2^)	42.0	(36.9–50.4)

Data are presented as medians (interquartile range)

Abbreviations: *Cr* creatinine, *eGFR* estimated glomerular filtration rate

**Table 4:** Pre- and postoperative serum creatinine levels and the estimated glomerular filtration rate

					*P*-value^*^
Serum Cr level (mg/dL)	Preoperative	1.23 (1.17–1.80)	Postoperative	1.18 (1.09–1.47)	0.496
eGFR (mL/min/1.73 m^2^)	Preoperative	39.0 (29.7–48.4)	Postoperative	42.0 (36.9–50.4)	0.45

Data are presented as medians (interquartile range).

Abbreviations: *Cr* creatinine, *eGFR* estimated glomerular filtration rate

^*^P-value based on the Mann–Whitney U test

**Fig. 1:**
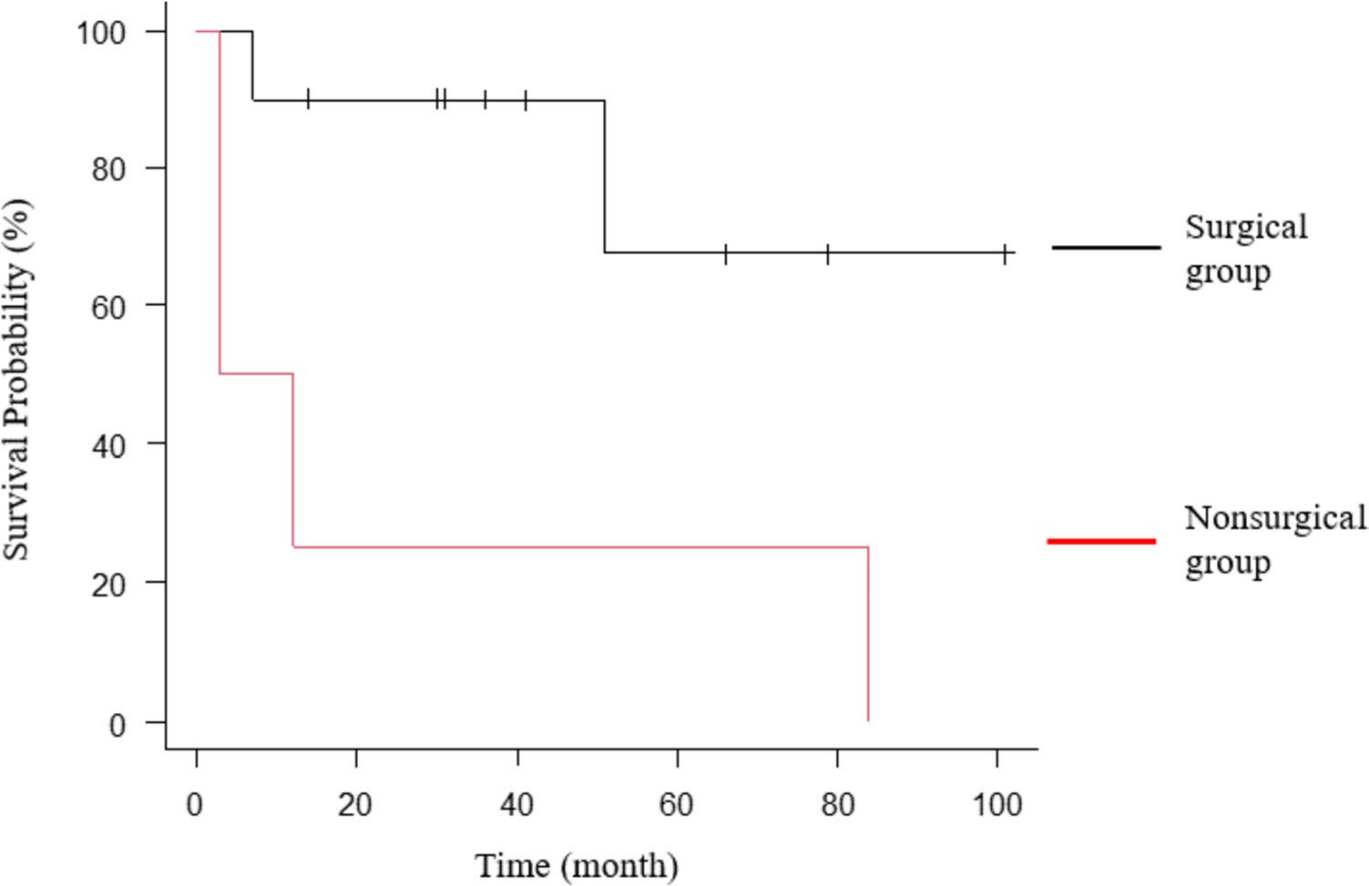
Kaplan–Meier estimate of the 5-year survival for the surgical and nonsurgical groups

The surgical group (*n* = 10) had a significantly better prognosis than the nonsurgical group (*n* = 4) (P = 0.02) (Fig. 1). Those patients treated with best supportive care were excluded from the nonsurgical group. The median length of follow-up was 33.5 months (range, 3–101 months) for all patients. Recurrence was identified in only one patient from the surgery group. None of the patients received postoperative adjuvant chemotherapy. Two patients without operative indications and those with stage IIIB or more advanced cancer received chemotherapy and radiation therapy. One of two patients with small cell lung cancer received first-line treatment (carboplatin plus etoposide) and second-line therapy (amrubicin monotherapy) without dose reduction. Although he completed chemotherapy without kidney failure, he died of cancer 12 months later. The other patient with small cell lung cancer was treated with palliative radiation for cancer-related pain; however, his liver metastatic lesion grew rapidly, and chemotherapy could not be performed. Thus, he received the best supportive care. Of two patients who had early-stage cancer but were treated with radiation therapy for comorbidities, one died of ischemic heart disease and the other died of radiation pneumonitis.

## Discussion

We reported the clinical course of 15 patients with lung cancer after kidney transplantation. The lung cancer was detected relatively early during follow-up after kidney transplantation, and curative surgery was performed in 10 of the 15 patients. There were no perioperative complications related to kidney transplantation and the prognosis of the surgical group was favorable.

The overall prognosis was worse for patients with lung cancer who received a solid organ transplant than for those who did not undergo transplantation [7]. Lung cancer after solid organ transplantation was associated with short survival despite surgical resection and radiation therapy. In an Italian multicenter study, the 5-year and 10-year survival rates of 18 patients with lung cancer after kidney transplantation among 3537 kidney transplant recipients were 32.9% and 0%, respectively [8]. Among 2793 patients who underwent kidney transplantation in China, 14 (0.5%) developed lung cancer, and 10 were treated surgically [9]. The overall 5-year survival rate after diagnosis of these 14 patients was 17.9%. The surgical group in our study had a relatively better prognosis because radical surgery was performed for early-stage lung cancer. Another study also found lung cancer surgery after solid organ transplantation to be associated with a high incidence of postoperative complications and a high 90-day postoperative mortality rate [10]. Pneumonia was a common postoperative complication and inflammatory respiratory complications were the only predictor of poor overall survival [11]. The incidence of wound complications can increase after kidney transplantation [12] and lung resection in immunosuppressed patients may also be associated with the risk of bronchopulmonary fistula and other complications. In a previous report from Japan, pneumocystis pneumonia developed in one patient, who required hemodialysis; however, the other patients did not suffer any serious complications [13]. In our study, there were no perioperative complications. Pericardial fat pad reinforcement was performed in most lobectomies; however, further studies are needed to determine its efficacy. In terms of operative methods, the percentage of wedge resections was high. Complete resection with wedge resection can be performed for early-stage small peripheral lung cancer [14]. Lobectomy and mediastinal lymph node dissection are the standard surgical methods for early-stage non-small-cell lung cancer in patients who have undergone kidney transplantation as well as in patients who have not undergone kidney transplantation. Segmentectomy is now considered the standard surgical procedure for patients with small peripheral non-small-cell lung cancer [15]. However, more data should be collected to establish the usefulness of sublobar resection in patients with lung cancer after kidney transplantation. Although none of the patients in the present study received adjuvant therapies, postoperative adjuvant therapy could be considered based on the pathological stage and genetic information as several important studies have been reported recently [16, 17].

At present, there is no consensus on cancer screening protocols, particularly for lung cancer, for patients who have received a solid organ transplant [18]. Yearly low-dose CT is recommended for adults aged 55–79 years who have smoked one pack a day for 30 years or equivalent (two packs a day for 15 years) [19]. Clinicians are often dependent on the clinical practice guidelines of regional and national transplant societies. Despite frequent medical and radiological examinations, lung cancer is usually diagnosed at an advanced stage, and its overall prognosis remains poor [20]. Routine examinations after kidney transplantation were performed based on our own protocol and several cases of early-stage lung cancers were identified. Notably, lung cancer was detected at an early stage in most patients, many of whom underwent curative surgery. Conversely, some patients already had advanced-stage lung cancer at the time of examination. Two patients with symptoms were diagnosed with advanced-stage cancer and had poor prognoses. Thus, routine follow-up after kidney transplantation is important to detect lung cancer before symptoms develop. As lung cancer is difficult to diagnose early, an optimal follow-up protocol must be established in the near future.

The long-term use of immunosuppressive agents accounts for the increased incidence of malignancy after kidney transplantation because immunosuppressive agents reduce the immune surveillance of tumor cells [21]. The Taiwan national database documented a 3.3-fold increase in the overall standardized incidence ratios after kidney transplantation [22]. This study evaluated the number of malignant tumors, including lung tumors that developed during the research period. Among 2593 patients who underwent kidney transplantation at our institution, 206 were found to have a collective 220 (7.9%) tumors, which is higher than in the previous report [23]. Post-transplant lymphoproliferative disorder (*n* = 33 patients) was the most common malignancy, followed by renal cancer (*n* = 26), skin cancer (*n* = 21), colorectal cancer (*n* = 19), lung cancer (*n* = 15), gastric cancer (*n* = 14), breast cancer (*n* = 14), prostate cancer (*n* = 13), liver cancer (*n* = 12), thyroid cancer (*n* = 11), pancreatic cancer (*n* = 9), urinary tract cancer (*n* = 8), uterine cancer (*n* = 8), tongue cancer (*n* = 3), pharyngeal cancer (*n* = 3), esophageal cancer (*n* = 3), duodenal cancer (*n* = 3), ovarian cancer (*n* = 2), brain tumor (*n* = 2), and carcinoma of unknown primary (*n* = 1). The true incidence of malignancy after kidney transplantation was challenging to assess accurately because of the long study period and loss of some patients to follow-up. Furthermore, our study had four immunosuppressant protocols, which varied according to the type of immunosuppressive agents used and other factors. Interestingly, the frequency of lung cancer after kidney transplantation in our study was almost the same as that in previous studies [8, 9].

This study has several limitations. First, it was retrospective in nature, so the true incidence of primary lung cancer in the recipients of kidney transplantation could be underestimated. Second, the small sample size of this single-center study did not allow for a multivariate analysis of patient survival. Hence, survival differences have not been interpreted.

In conclusion, routine thoracic examination is recommended for the early detection and treatment of lung cancer in patients who have undergone kidney transplantation. There were no perioperative complications related to thoracic surgery in any of the kidney transplant recipients. Furthermore, radical surgery for early-stage lung cancer may improve the prognosis.

## Data Availability

Data supporting the findings of this study are available from the corresponding author upon reasonable request.
